# First case of chronic wasting disease in Europe in a Norwegian free-ranging reindeer

**DOI:** 10.1186/s13567-016-0375-4

**Published:** 2016-09-15

**Authors:** Sylvie L. Benestad, Gordon Mitchell, Marion Simmons, Bjørnar Ytrehus, Turid Vikøren

**Affiliations:** 1 Norwegian Veterinary Institute, P.O. Box 750 Sentrum, 0106 Oslo, Norway; 2 Canadian Food Inspection Agency, National and OIE Reference Laboratory for Scrapie and CWD, Ottawa Laboratory Fallowfield, Ottawa, ON Canada; 3 Department of Pathology, Animal and Plant Health Agency, Woodham Lane, Addlestone, Surrey, KT15 3NB UK; 4 Norwegian Institute for Nature Research (NINA), P.O. Box 5685 Sluppen, 7485 Trondheim, Norway

## Abstract

Chronic wasting disease (CWD) is a fatal contagious prion disease in cervids that is enzootic in some areas in North America. The disease has been found in deer, elk and moose in the USA and Canada, and in South Korea following the importation of infected animals. Here we report the first case of CWD in Europe, in a Norwegian free-ranging reindeer in Southern Norway. The origin of the disease is unknown. Until now a low number of cervids, and among them a few reindeer, have been tested for CWD in Norway. Therefore the prevalence of CWD is unknown.

**Electronic supplementary material:**

The online version of this article (doi:10.1186/s13567-016-0375-4) contains supplementary material, which is available to authorized users.

## Introduction, methods, and results

Chronic wasting disease (CWD) is a fatal neurodegenerative disorder that affects cervids. CWD belongs to a group of prion diseases, which include scrapie in small ruminants, bovine spongiform encephalopathy (BSE) in cattle and Creutzfeldt-Jakob disease in humans.

The development of the disease is associated with the misfolding of a host-encoded prion protein, (PrP^C^) into resistant conformers (PrP^res^ also called PrP^CWD^ in cervids) which accumulate in the central nervous system, and ultimately results in death of the animal.

CWD has been diagnosed in captive and free-ranging cervids in 24 American states and two Canadian provinces [1].

It affects different species in the family *Cervidae*, mule deer (*Odocoileus hemionus*), white-tailed deer (*Odocoileus virginianus*), elk (*Cervus canadensis*) and moose (*Alces alces*) [2–4]. In addition, CWD has been diagnosed in red deer (*Cervus elaphus*) and sika deer (*Cervus nippon*) in South Korea as the result of importing CWD-infected elk from North America [5].

The clinical signs of CWD have mainly been observed in captive animals and are subtle and vary from case to case [6]. The first clinical signs are nonspecific and include variable behavioral changes such as listlessness, isolation from the herd, lowering of head and ears, repetitive walking and hyperexcitability, followed by weight loss. Other signs are polydipsia/polyuria, hypersalivation, grinding of the teeth, frequent regurgitation and difficulty swallowing. Secondary aspiration pneumonia may occur and stress can trigger the appearance of clinical signs and may lead to unexpected mortality. Diseased animals do not lose their appetite and often their rumens are filled with food, sometime with an excessive amount of water and sometimes dry. The duration of clinical disease varies among individuals, ranging from weeks to months.

Experimentally, CWD is transmissible to a range of cervids [7, 8]. In addition, a large spectrum of mammals are shown to be susceptible to CWD by intracerebral inoculation, like sheep, goats, cattle, cats, some rodents species (reviewed in [9]) and a series of transgenic mice carrying the PrP gene of different species [10]. The experimental transmission of CWD to non-cervid species by oral challenge is far less efficient, indicating a high species barrier under natural conditions. Interestingly, reindeer (*Rangifer tarandus tarandus*) have been shown to be susceptible to experimental oral transmission [11] while the North American subspecies of reindeer (*R. tarandus*) known as caribou, have not been diagnosed with CWD.

Four cervid species are prevalent in free-ranging populations in Norway: moose (*Alces alces*), red deer, roe deer (*Capreolus capreolus*), and reindeer. Norway harbors what is considered as the last remnants of wild tundra reindeer (*Rangifer tarandus tarandus*) in Europe, with approximately 25 000 animals in the winter population. The species is found in fragmented sub-populations in the remote alpine regions of Southern Norway. Most of the sub-populations make short-range seasonal migrations between different pasture areas and congregate in large herds in periods of the year. Here we report the first case of CWD in Europe in a free-ranging Norwegian reindeer.

A herd of approximately 400 free-ranging reindeer from the Nordfjella (Southern Norway) sub-population was approached by helicopter on March 2016. This was done in an attempt to chemically immobilize an individual animal to place a radio-collar, as part of a larger study addressing the spatiotemporal interactions between reindeer and humans. The CWD reindeer described in this report was found as the research crew searched for a missing dart in an area not far from where the herd first was approached. Judging by the tracks in the snow, this individual had split off from the main herd and run downhill, at first leaving a normal track, but afterwards leaving tracks suggestive of dragging one of its right limbs. The female reindeer was found recumbent, but did still have eye reflexes and could move its limbs. It had small amounts of white froth on its lips, a raised body temperature of 41.9 °C and it died shortly after. Because the case reported here involved an animal who died of natural causes, ethics approval is not required.

Based on dental eruption and wear, the reindeer was an adult over 2.5 years old, probably in the range of 3–4 years. Necropsy revealed that the female reindeer was not pregnant and was in below average body condition, weighing 43 kg and having only small amounts of body fat. It had small areas of hair loss on the chest, the elbows and the thighs. The main gross findings were multiple hemorrhages and ruptures in the skeletal muscles most prominent in the hind quarters, general congestion, and lung edema with areas of consolidation in the right cranial lung lobe. Tissue samples of brain, heart, lung, tracheobronchial lymph nodes, liver, kidney and skeletal musculature were fixed in 10% buffered formalin, routinely processed and embedded in paraffin before cutting sections (5 µm) that were stained with haematoxylin-eosin (HE) for histological examination.

**Figure 1:**
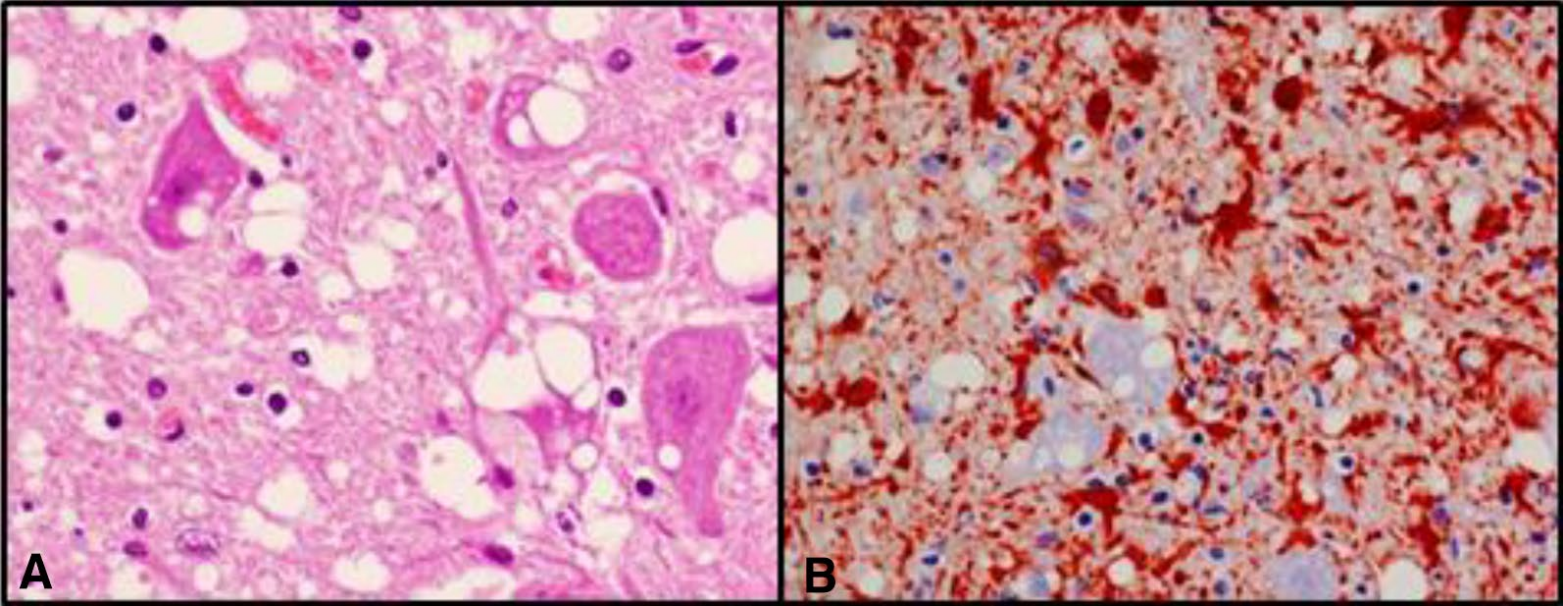
**Histology of the obex. A** HE staining showing vacuoles in the neurons and neuropil ×600. **B** Glial fibrillary acidic protein (GFAP) immunolabelling showing strong proliferation of reactive astrocytes (gliosis) in the obex area ×400.

Histology revealed acute degeneration of skeletal musculature and acute multifocal catarrhal bronchopneumonia with small amounts of foreign material in the inflamed lung tissue. In the brain, histopathological examination of the obex revealed vacuolation, especially in the dorsal motor nucleus of the vagus nerve (DMNV) (Figure 1A), in the neuropil and in neurons. However, the brain tissue showed a mild to moderate degree of autolysis which precluded an accurate description of the spongiform degeneration.

The medulla oblongata of this animal was analyzed by using a commercially available Enzyme-Linked Immunosorbent Assay test for detection of abnormal prion protein (PrP^res^) (TeSeE ELISA Bio-Rad) according to the manufacturers’ instructions. After an initial positive result, six other samples taken from different areas of the medulla and one from the cerebral cortex were also analyzed and found to be strongly positive (OD values above 2.9).

**Figure 2:**
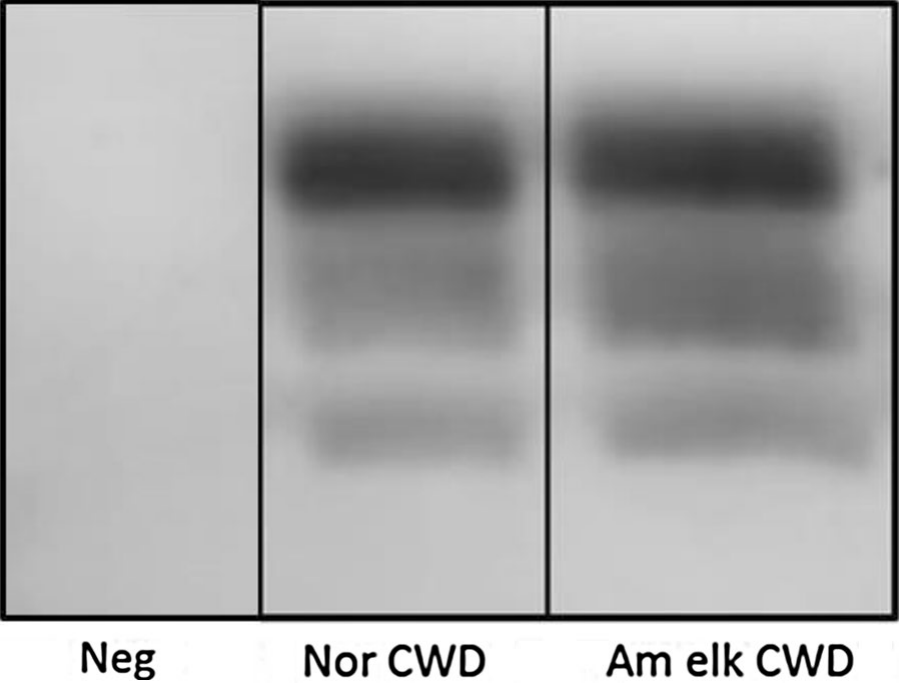
**Western blot detection of PrP** ^**res**^ **with TeSeE Western blot kit (Bio-Rad), using SHa31 and P4 mAb.** The Norwegian reindeer sample (Nor CWD) showed a typical 3-band pattern similar to the American elk CWD sample (Am elk CWD). No signal was seen in the negative sheep brain sample (Neg).

The presence of PrP^res^ was further demonstrated by a commercially available Western blot test (TeSeE Western blot, Bio-Rad) using the monoclonal antibodies SHa31 (AbI kit reagent) together with an additional monoclonal antibody, P4 at a dilution of 1:1000. The medulla sample of the reindeer displayed a proteinase K (PK)-resistant 3-bands pattern between 17 and 29 kDa, identical to patterns from an American elk CWD control (kindly provided by Terry Spraker, CSU Vet Diagnostics Lab, CO, USA) (Figure 2).

The distribution of PrP^CWD^ was visualized in the brain tissues and in two tracheobronchial lymph nodes by immunohistochemical labelling using the antibodies F89/160.1.5 and 2G11 which bind to residues 142–145 and 149–161 of ovine PrP respectively. Briefly, tissue sections on poly-l-lysine glass slides were deparaffinised, rehydrated, treated in formic acid 98% for 30 min and boiled by hydrated autoclaving at 121 °C in 0.01 M citric acid, pH 6.1 for 30 min. A commercially available kit (EnVisionTM + System HRP (AEC) DAKO, Glostrup, Denmark) was applied with a mixture of mAb F89/160.1.5 and 2G11 at a dilution of 1:700 and 1:200 respectively for 30 min at 37 °C.

**Figure 3:**
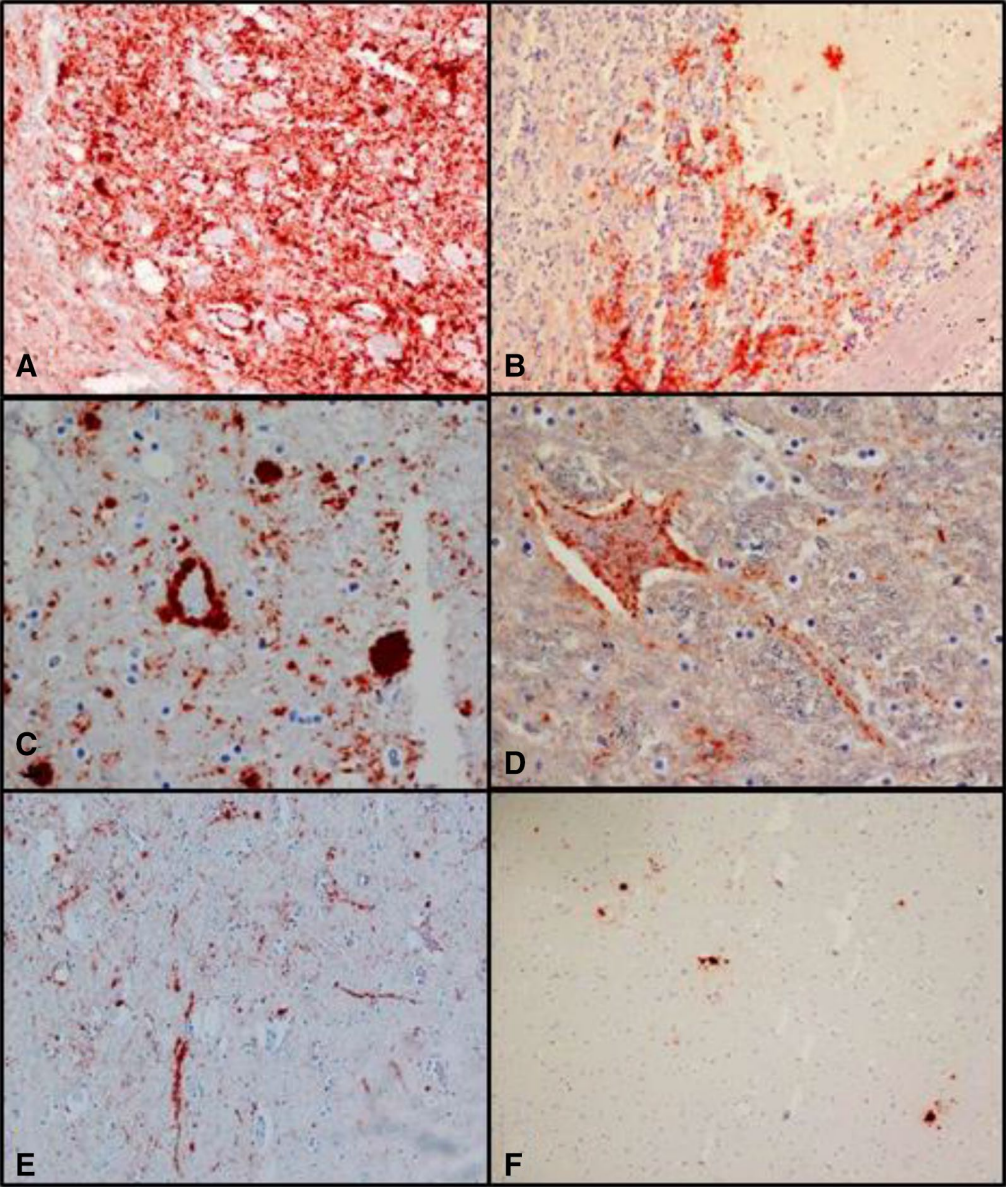
**IHC labelling of PrP** ^**CWD**^ **using F89/160.1.5 and 2G11 mAb. A** Obex, intense coarse particulate labelling in the DMNV ×200. **B** Cerebellum, patchy labelling in the granular layer and stellate in the molecular layer ×200. **C** Ventral midbrain, scattered granules or accumulations of PrP^CWD^ that appear as plaques ×400. **D** Ventral midbrain, neuronal and axonal labelling ×400. **E** Ventral midbrain, linear type labelling ×100. **F** Dorsal midbrain, sparse immunolabelling, plaque-like accumulation of PrP^CWD^ ×100.

The obex region showed a strong particulate coalescing, perineuronal labelling of PrP^CWD^ especially in the DMNV. Less severely affected nuclei included the spinal tract of the trigeminal nerve, hypoglossal nucleus and the area postrema, and some labelling was seen in the cuneate nucleus, medial lemniscus and the olivary nuclei. The PrP^CWD^ labelling in the cerebellum was less prominent, with patchy labelling in some areas, often heavier in the granular layer, and moderate in the form of stellate labelling reminiscent of astroglial processes in the molecular layer. The remaining regions of the brain displayed PrP^CWD^ labelling (coarse, patchy, stellar, plaque-like, perivascular, linear or intraneuronal) as illustrated in Figure 3. Labelling was more prominent in the ventral regions of the brain. Marked PrP^CWD^ immunolabelling was present in the lymphoid follicles of the tracheobronchial lymph nodes.

For labelling of astrocytes, the glial fibrillary acidic protein (GFAP), the EnVisionTM + System HRP (AEC) DAKO, Glostrup, Denmark kit protocol was applied using polyclonal anti-GFAP antibody (DAKO Z 334 Glostrup, Denmark) at a dilution of 1:2000 for 30 min. The obex showed marked immunolabelling (Figure 1B).

Genomic DNA was extracted from frozen brain and the prion protein gene (*PRNP*) open reading frame was amplified and sequenced (Applied Biosystems 3500×L genetic analyzer with Big Dye Terminator chemistry). The *PRNP* sequence of this reindeer was identical to that of the two reindeer that were previously found to be susceptible to CWD by experimental oral infection (homozygous V (valine), G (glycine), S (serine) and V at codons 2, 129, 138 and 169) [11].

## Discussion

This case of CWD in a free-ranging Norwegian reindeer is the first report of naturally occurring CWD in Europe. The disease was detected through the routine CWD testing of cervids older than 18 months that are necropsied at the Norwegian Veterinary Institute. Other pathological findings were consistent with acute exertional (capture) myopathy and small areas of aspiration pneumonia, both probably secondary to CWD. Exertional myopathy is associated with physiological imbalances following extreme exertion and stress, and it is reported that deer with subclinical or early clinical CWD are susceptible to sudden death after stressful situations [6]. Aspiration pneumonia is well documented in animals suffering from CWD and difficulty in swallowing and a flaccid esophagus with reduced muscular tone have been suggested as possible explanations [4]. This reindeer was not emaciated, but she was in poor body condition as compared to a healthy non-pregnant female reindeer in late winter. In conclusion, based on the case history and the relatively widespread distribution of PrP^CWD^ in the brain, there is reason to conclude that the animal had a spongiform encephalopathy compatible with CWD at an early clinical stage.

The presence of two different strains has been demonstrated in North American CWD isolates when passaged into ferrets [12], Syrian hamsters [13] and transgenic mice with cervid PrP [14]. The strains are designated differently (CWD-WI and CWD-CSU, Sgha CWDmd-f and Sgha CWDmd-s, or CWD1 and CWD2) in ferrets, Syrian hamsters and mice, respectively. Each “pair” comprises a strain transmitting disease after a short incubation time and providing a widespread distribution of PrP^res^ in the CNS, and a second strain with a longer incubation time and more limited deposition of PrP^res^. Notably, there are no differences in the biochemical glycoprofile between the two strains, which makes it impossible to distinguish them without bioassays [14]. The Norwegian CWD reindeer isolate had a similar glycoprofile to the American elk CWD sample (Figure 2) and Canadian CWD isolates used as controls for the Western blot analysis (Additional file 1), including reindeer orally inoculated with CWD material [11]. The immunohistochemical distribution of PrP^CWD^ in the obex, the cerebellum and the lymph nodes by IHC was also comparable to those found in Canadian reindeer successfully orally challenged with CWD. Transmission studies have now been initiated on a panel of transgenic mice to characterize the Norwegian reindeer isolate.

A major question concerns the origin of CWD in Norway. Importation of CWD infected deer could be the source of infection, as was the case in South Korea. However, Norway has strict legislation and enforcement regarding the importation of live animals and importation of cervids is not allowed. The only exceptions are a few moose that have been imported to zoos from Sweden. All red deer (*Cervus elaphus*) kept in farms originate from wild Norwegian populations. We find it unlikely that this CWD case is due to imported infected cervids unless illegal imports from North America to Norway have taken place. It is however noteworthy that Finland’s white tailed deer population, estimated at 60 000 animals, originated from one import from North America in 1934 of four does and one buck. Due to the CWD situation in North America, Finland targeted TSE testing especially to the white tailed deer in the period between 2003 and 2015, and a total of 643 white tailed deer have been tested and found negative for CWD (Sirkka-Liisa Korpenfelt, EVIRA, Finland, personal communication).

Another possibility of contamination is through hunting urine baits imported from North America, but for the time being, no information about the use of these baits in Norway is available.

It has been speculated that the origin of CWD in North American cervids may be associated with classical scrapie because some scrapie infected sheep had been penned together with deer at a research center between 1968 and 1971 [4, 15, 16]. To support this hypothesis, Tamgüney [17] reported the successful transmission of one classical scrapie isolate into transgenic mice carrying the elk prion protein gene [Tg(ElkPrP) mice], but it is noteworthy that the agent signature (as defined by lesion profile) in these mice was different from that in mice inoculated with CWD, suggesting that the scrapie and CWD agent were distinct strains. Norway has had a scrapie surveillance program in place since 1997 with a total of 264 000 small ruminants analyzed. Few cases of classical scrapie have been diagnosed in Norway and the last case was identified in 2009. There are no reports of classical scrapie within the range of the Nordfjella reindeer sub-population, but as sheep traditionally are transported over long distances to graze in mountain pastures, it cannot be formally excluded that reindeer in this or a nearby subpopulation could have been exposed to sheep with classical scrapie at some point of time.

In contrast to classical scrapie is Nor98/atypical scrapie diagnosed in sheep in Norway. Between five and 13 cases have been identified each year the past 10 years, and they are found over the whole country, including where the CWD reindeer was discovered. Whether Nor98/atypical scrapie could be the source of CWD in Norway cannot be excluded, despite the fact that, as it could be expected in case of direct transmission, the distinctive molecular signature of Nor98/atypical scrapie, a multiband WB pattern [18] was not observed in the present reindeer.

A plausible alternative to the occurrence of CWD in Europe could be that a cervid developed a genetic or spontaneous TSE which subsequently spread horizontally to other cervids. As cervids may eat or gnaw on remnants of carcasses, we may speculate that there could have been an incident more or less analogous to the Kuru epidemic on Papua New Guinea, which is suggested to have started with ritual cannibalism of an individual with sporadic Creutzfeldt-Jakob Disease [19].

Currently we have no information about the distribution and prevalence of CWD in the Norwegian cervid population. Whether CWD is contagious among reindeer is also an open question, but should be expected. The social behavior of reindeer living close together in herds and grazing on the ground may increase the likelihood for CWD transmission. As opposed to most of the prion diseases, infectivity in cervids is demonstrated in many peripheral tissues, such as muscles [20], elk antler velvet [21], endocrine glands [22] and in excreta like urine, saliva, blood and feces [23–26]. In the present reindeer, PrP^CWD^ was detected by IHC in the tracheobronchial lymph node. This vast distribution of infectivity in the host, together with a high stability of prions in the environment [27–29] can explain why CWD is known as the most contagious prion disease, with a prevalence of up to 30% in free-ranging herds or 90% in captive herds [6, 30] and we should be prepared for detecting additional CWD cases in the Norwegian cervid population.

Norway has a particular responsibility to manage and protect the last remnant of the wild tundra reindeer in Europe, and the detection of CWD in one of these sub-populations is of great concern. Efforts are being made to reduce the migration of reindeer from Nordfjella to neighboring sub-populations and known migration paths are being monitored. Measures such as intensified nationwide CWD surveillance are being planned by the Norwegian Food Safety Authority and the Norwegian Environment Agency.

Surveillance for CWD in Europe has been limited. In Norway, approximately 2200 cervids have been tested from 2004 to 2015, of which only ten were free-ranging reindeer, but none were from the Nordfjella area. The small number of wild reindeer tested reflects that the chances of a sick animal in such remote areas being observed, reported and submitted for testing are small. The European Food Safety Authority (EFSA) stated that the occurrence of CWD could not be excluded in cervids in Europe, “especially in remote and presently unsampled areas” (EFSA journal 2010). Because of the limitation of the surveillance program in cervids, it is not possible to exclude that CWD has been present in Norway or Europe for decades without being detected until now. This assumption is strengthened by the discovery, at the time of writing of this paper, of two additional CWD cases in moose in Norway, originating from a region situated about 300 km from the Nordfjella area where the CWD infected reindeer was found. The prevalence, epidemiology and implications of CWD in Norwegian and European cervids remain to be determined.

CWD is enzootic in multiple regions in North America and unfortunately the disease is spreading. To our knowledge, this is the first case report of CWD in Europe and the first case of the disease naturally occurring in reindeer worldwide. The origin, prevalence, and incidence of CWD in Norway are currently not known, but being investigated.

## Additional file

10.1186/s13567-016-0375-4 **Western blot detection of PrP** ^**res**^ **with TeSeE Western blot kit (Bio-Rad), using SHa31 mAb, upper figure, or P4 mAb, lower figure.** A typical di-, mono- and unglycosylated protein banding pattern characteristic of CWD in cervids, was observed in the homogenate prepared from the Norwegian reindeer (16TSE96). Comparable molecular weight and glycoform ratios were found in the North American positive controls from different cervid species (white-tailed deer, elk, red deer), including experimentally infected reindeer (reindeer #s 12, 47 and 112) [11].

## Abbreviations

CWD: chronic wasting disease
BSE: bovine spongiform encephalopathy
PrP^C^: host-encoded prion protein
PrP^res^: resistant prion protein, also called PrP^CWD^ in cervids
HE: haematoxylin-eosin
DMNV: dorsal motor nucleus of the vagus nerve
PK: proteinase K
*PRNP*: prion protein gene
V: valine
G: glycine
S: serine
Tg(ElkPrP) mice: transgenic mice carrying the elk prion protein gene
EFSA: European Food Safety Authority
