# Supplementary figures and images for: First case of chronic wasting disease in Europe in a Norwegian free-ranging reindeer
Source: Vet Res. 2016 Sep 15;47:88. doi: 10.1186/s13567-016-0375-4 (PMC5024462; doi:10.1186/s13567-016-0375-4)

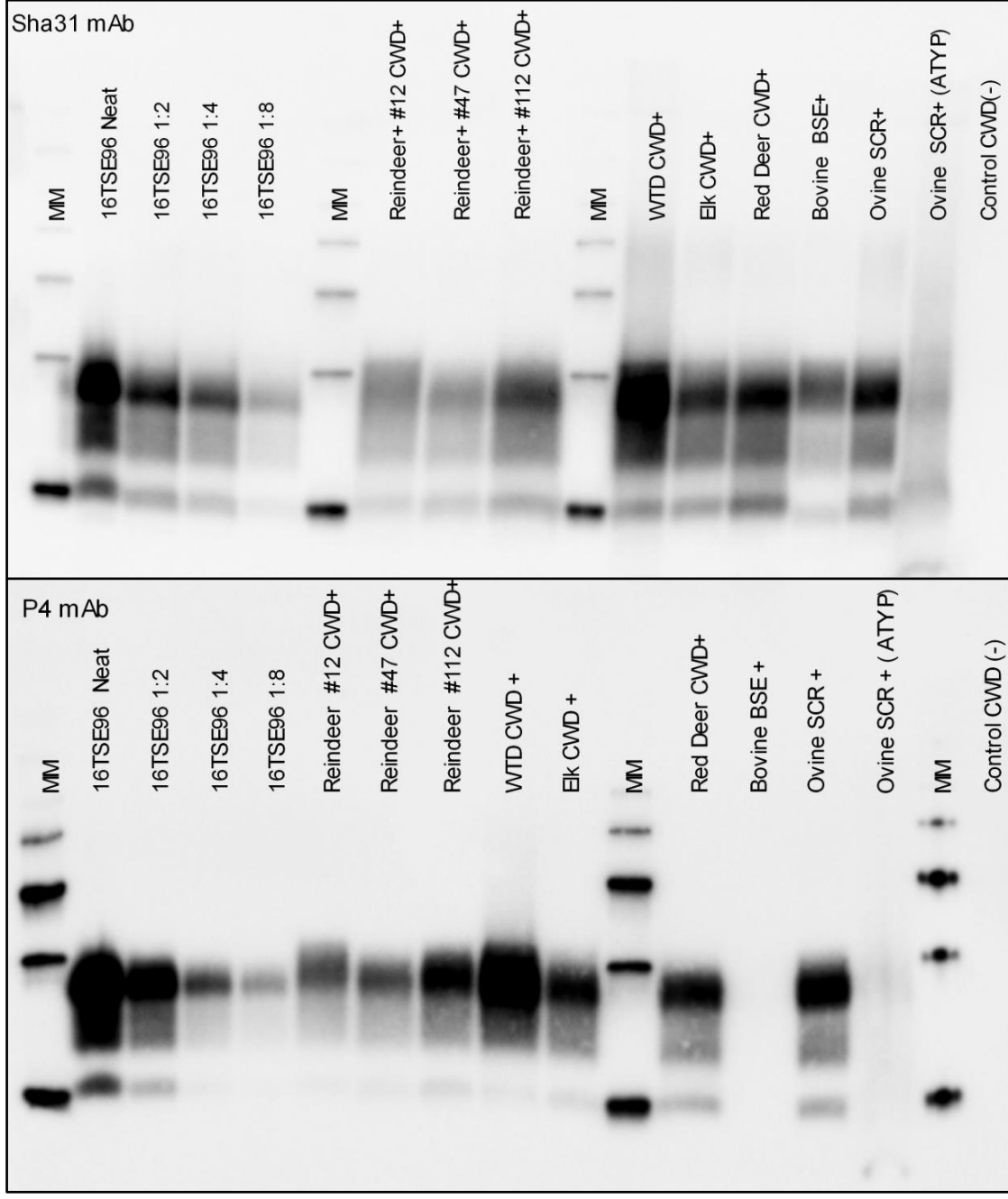

Supplement: Supplementary file 1 — 10.1186/s13567-016-0375-4 Western blot detection of PrP res with TeSeE Western blot kit (Bio-Rad), using SHa31 mAb, upper figure, or P4 mAb, lower figure. A typical di-, mono- and unglycosylated protein banding pattern characteristic of CWD in cervids, was observed in the homogenate prepared from the Norwegian reindeer (16TSE96). Comparable molecular weight and glycoform ratios were found in the North American positive controls from different cervid species (white-tailed deer, elk, red deer), including experimentally infected reindeer (reindeer #s 12, 47 and 112) [11]. [file 13567_2016_375_MOESM1_ESM.pdf]
